# Earthquake-induced soil landslides: volume estimates and uncertainties with the existing scaling exponents

**DOI:** 10.1038/s41598-023-35088-6

**Published:** 2023-05-19

**Authors:** Ali P. Yunus, Chen Xinyu, Filippo Catani, Srikrishnan Siva Subramaniam, Xuanmei Fan, Dou Jie, K. S. Sajinkumar, Ankita Gupta, Ram Avtar

**Affiliations:** 1 Department of Earth and Environmental Sciences, Indian Institute of Science Education and Research Mohali, Mohali, Punjab 140-306 India; 2 Faculty of Environmental Earth Science, Hokkaido University, Sapporo, 060-0810 Japan; 3 Department of Geosciences, University of Padova, 35131 Padova, Italy; 4 Centre of Excellence in Disaster Mitigation and Management, Indian Institute of Technology Roorkee, Roorkee, Uttarakhand 247667 India; 5 State Key Laboratory of Geohazard Prevention and Geoenvironment Protection, Chengdu University of Technology, Chengdu, People’s Republic of China; 6 Three Gorges Research Center for Geohazards, The China University of Geosciences, Wuhan, 430074 People’s Republic of China; 7 Department of Geology, University of Kerala, Thiruvananthapuram, Kerala 695 581 India

**Keywords:** Hydrology, Natural hazards

## Abstract

Quantifying landslide volumes in earthquake affected areas is critical to understand the orogenic processes and their surface effects at different spatio-temporal scales. Here, we build an accurate scaling relationship to estimate the volume of shallow soil landslides based on 1 m pre- and post-event LiDAR elevation models. On compiling an inventory of 1719 landslides for 2018 M_w_ 6.6 Hokkaido-Iburi earthquake epicentral region, we find that the volume of soil landslides can be estimated by γ = 1.15. The total volume of eroded debris from Hokkaido-Iburi catchments based on this new scaling relationship is estimated as 64–72 million m^3^. Based on the GNSS data approximation, we noticed that the co-seismic uplift volume is smaller than the eroded volume, suggesting that frequent large earthquakes (and rainfall extremes) may be counterbalancing the topographic uplift through erosion by landslides, especially in humid landscapes such as Japan, where soil properties are rather weak.

## Introduction

Strong seismic shaking in a steep mountain belt induces hundreds to thousands of landslides, thereby eroding the topography instantaneously^1^. Quantifying the volume of earthquake-induced landslides (EQIL) is critical to understand the topographic evolution and mountain building processes^2^, mass wasting and sediment budget^3,4^, reservoir siltation^5^, to ascertain chains of geohazard risk in downstream patches including debris flows and floods^6,7^, vegetation dynamics and carbon sink^8–10^, and other atmospheric and surface processes^11^. However, only a limited number of studies succeeded in accurately quantifying EQIL volumes immediately following a seismic event^12^. A number of reasons may explain the paucity of landslide volume studies: (1) the three-dimensional nature of the object landslide that is, conversely, intrinsically mapped as two-dimensional, (2) inaccessible terrains, (3) impractical to quantify huge debris piles in field measurements, (4) lack of high-resolution pre-event topographic information, and (5) total and partial reactivations of landslides that may merge sediment volumes pertaining to different triggering events^13,14^.

There exist several practical examples of estimating landslide volumes that rely on empirical power-law relationships between the area of a landslide and its volume^2,15,16^Although area (A) can be easily obtained from a landslide inventory map, volume (V) typically requires a small amount of field component or accurate pre- and post-event topographic data to finally compute the scaling exponent γ and intercept α in the equation V = α.A^γ^^17^. Literature suggests that the major source of uncertainty in the volume estimation comes from the uncertainty in the parameter γ^2,18^. The value of γ is reported to be in the range of 1.0 ≤ γ ≤ 1.9 for a volume sizes of 10^2^ m^3^ to 10^3^ m^3^^19^.

Empirical models for volume estimation originated four decades ago^17^, but the large uncertainties arising from the material differences and the lack of validation data caused researchers to be cautious while employing the results for practical use. Significant errors in the early scaling relationship may be accounted for by the underdeveloped remote sensing and GIS technologies at that time^20^. Before the availability of multi-temporal high-resolution digital elevation models (DEM), reliable scaling relationships were therefore dependent on the possibility to execute intensive and expensive field surveys. Despite the difficulty, Imaizumi et al.^21^ measured 51 rainfall-induced landslide scars in the field with sizes ranging from 10 m^2^ to 3000 m^2^, and came up with a scaling exponent of γ = 1.31. But in the case of EQIL, the number and size of landslides are much larger, and hence the difficulties in executing an accurate field measurement are often overwhelming. Regardless of their high accuracy, conventional measurements in field surveys are limited to landslide sizes of some extent, mainly owing to inaccessibility to climb-up to the scar regions in mountain terrains.

With the advancement in GIS and Earth Observation technologies, more scaling relationships based on remote measurements have been proposed. Massey et al.^12^ analyzed a catalogue of 17,256 landslides triggered by the M_W_ 7.8 Kaikōura, earthquake, New Zealand using DEMs generated from stereo aerial photographs, and found a scaling exponent γ = 1.109 for all the landslides and 1.13 ± 0.03 for soil landslides alone. Nevertheless, the classification of the debris into different material types and units yielded different scaling exponents, suggesting that different scaling exponents are needed for soil, bedrock, and mixed (soil–bedrock) landslides.

Researchers have noticed regional differences in scaling relationships for soil landslides, depending on the thickness of the regolith^22^. Larsen et al., (2010) reported that the lowest γ-values are found for soil landslide in glaciated and semi-arid regions, where soil formation is thin compared to the thick soil layers to be found in temperate and tropical regions. In loess depsoits, for instance the landslides in Chinese Loess Plateau, the γ-values are found similar to soil landslides found in tropical and temperate region because of thick layering^23^.For such reasons, it is imperative to develop accurate landslide volume estimates for each earthquake inventory. Moreover, the peculiar topography of Iburi in Hokkaido prefecture, comprising multiple tephra layers some several meters deep, is worthy of attention.

In this paper, we develop a volume-area relationship for landslides triggered by the 2018 M_w_ 6.6 Hokkaido Eastern Iburi Earthquake derived from high-resolution LiDAR DEMs. Using the scaling relationship, we quantify the total volume of eroded materials from the hillslopes of the entire affected areas. As far as the authors knowhow, the selected study site is probably the only case where EQIL debris are completely or near completely moved out of hillslopes because of their low relief and gentle topography; hence, the area-volume relationship presented in this work can be useful globally for accurate soil-landslide volume estimates. In addition, we investigate the uncertainties in existing scaling relationships and their influence on total volume estimates. From these results, we briefly discuss how large earthquakes may counterbalance the topographic uplift in terms of net rates of sediment volume change.

In tectonically active areas, thrust faulting earthquakes progressively increases the steepness and height of mountain ranges^24,25^. For example, Mw 7.8 Gorkha earthquake generated about 1 m of uplift in the Kathmandu Basin, which is overall one order of magnitude higher than the erosional processes by landslides^26^. However, a few studies have demonstrated the impact of large earthquakes on high magnitude topographic erosion through landslides, raising fundamental questions concerning orogenic processes. For instance, analysis of EQIL data from Mw 7.9 Wenchuan shows that this earthquake event dominates the erosional budget^3^. We therefore aim to estimate the net volume balance associated to the 2018 Hokkaido-Iburi earthquake from landslide volume analysis. This is important because it also reveals how different magnitude earthquakes behave in different topographies in terms of erosion and uplift, and can contribute to the overall understanding on the mountain building processes.

## Materials and methods

**Figure 1:**
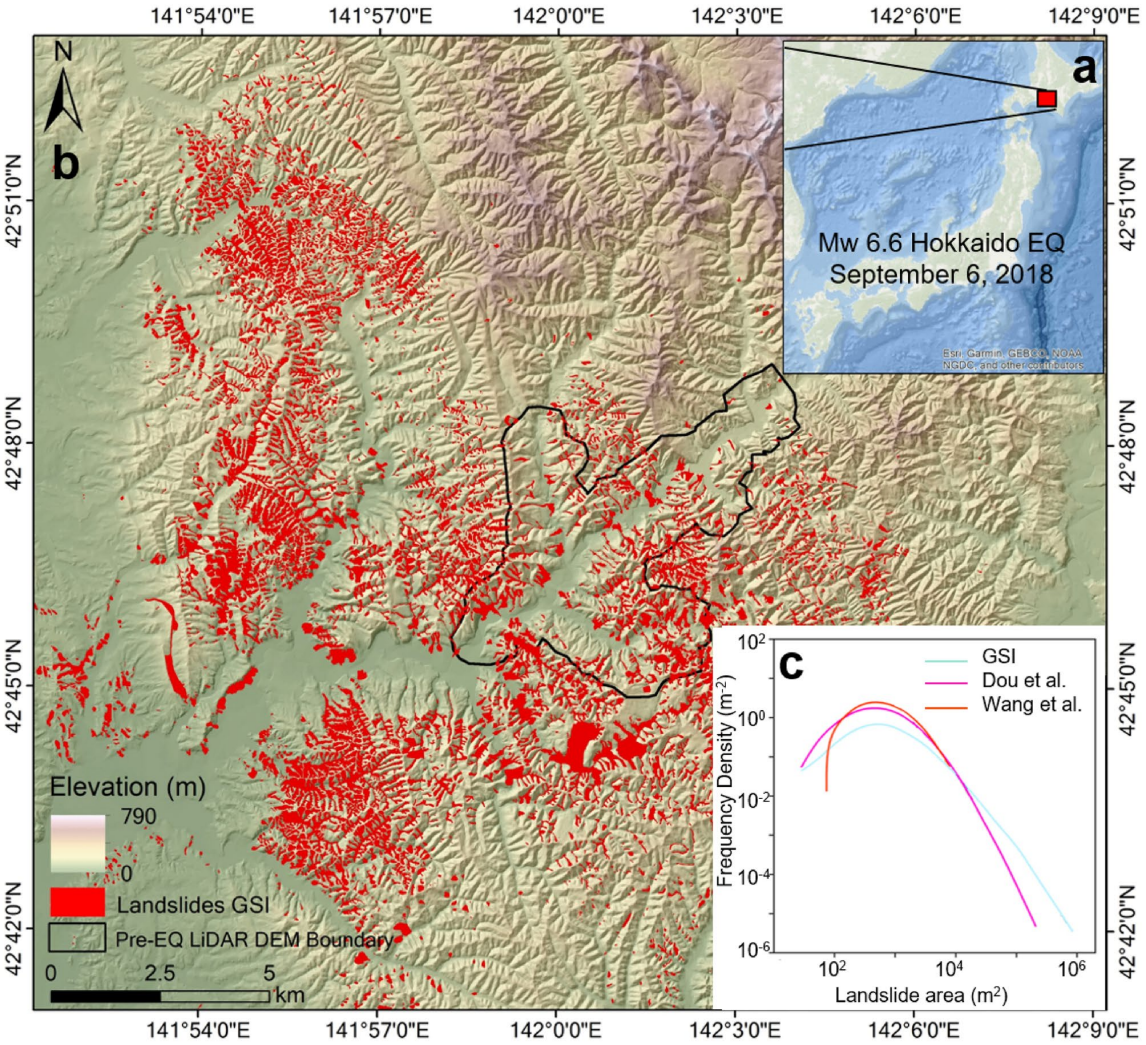
(**a**) Location map of Hokkaido Eastern Iburi M_w_ 6.6 earthquake epicentral area, (**b**) showing the location of landslides mapped by GSI (red shaded areas;). Caliberation area, i.e., the areal extent of 1 m pre and post event DEM availability are shown in black polygon. (**c**) shows the frequency density-landslide area plot for three different inventories (GSI—blue line, Dou et al. 2020—pink line, and Wang et al. 2019—orange line).

The September 6, 2018 Hokkaido Eastern Iburi M_w_ 6.6 (M_j_ 6.7) earthquake triggered about 10,120 landslides^27^, mostly shallow soil slides, spanning a geographical area of ~ 500 km^2^ (Fig. 1). Following the earthquake, several landslide inventories were created for this event with the landslides number ranging from three thousand to more than ten thousand failure surfaces^27–31^. The large variation in the number of landslides mapped in different inventories is due to the difference in the mapping criteria adopted. For example, the Geospatial Information Authority of Japan (GSI), manually mapped the landslides from 0.5 m aerial photos and their inventory contains 3307 landslide polygons; this number is much fewer than other inventories, because GSI combined several small landslides in the same slope aspect into a large single landslide. Wang et al. (2018) digitized 7941 landslide polygons by comparing pre- and post-event 3.7 m resolution Planet images, and Dou et al. (2020), manually delineated 10,120 landslide polygons based on 0.5 m aerial photos and 2 m DEM for avoiding the shadow effect, and further to separate many clustered landslides into several smaller ones (see Supplementary Fig. S1). Accordingly, the total landslide area varies from 26.07 km^2^^28^to 27.97 ^27^ to 48.77^28^ km^2^.

Completeness of mapping assessment for these three inventories was verified by searching for landslides in the neighboring zones using large swath images collected by Landsat 8 OLI and Sentinel-2 satellite after the earthquake. On the other hand, mapping accuracies varies from interpreter to interpreter; but avoidance of non-coseismic landslides is achieved by performing manual cross-checking with pre-earthquake images. Since there is a large difference in both the number and area of landslides, we used all the three aforementioned open inventories to approximate the total volume of materials eroded from the study area catchments and to quantify the uncertainties in landslide mapping.

Surface geology in the study area is comprised of volcanic pumice fall deposits from Tarumae (~ 9000 years ago), Eniwa (~ 20,000 years ago), and Shikotsu (~ 46,000 years ago) volcano, having a soil thickness of 0.85 m to 2.65 m^32,33^. Osanai et al. (2019) reported that the topsoil layer in the middle of the failed slope reaches a thickness of 2.5–3.5 m. In places, the volcanic soil deposits are even deeper than 8 m^34^. Additionally, the data from global 1-km gridded thickness of soil, regolith, and sedimentary deposit layers^35^ show an average thickness of 2.93 m in the calibration region.

High resolution (1 m) LiDAR DEMs for pre-earthquake and post-earthquake periods were obtained from the Hokkaido Provincial Government. The pre-earthquake LiDAR DEM was developed for the purpose of Atsuma Dam Construction in 2012 by the Construction Management Department of the Atsuma district in the EPSG2454 JGD2000 coordinate system. Following the earthquake in 2018, the Forestry Division of the Atsuma district carried out a survey for obtaining the post-event LiDAR DEM in the same coordinate system. While the total area covered in the pre-earthquake survey was only 29.41 km^2^ (hereafter refer as calibration area), the post-earthquake survey was about 512 km^2^ (hereafter refer as testing area), i.e., ~ 95% of the total earthquake-affected area. The accuracy of both DEMs has been evaluated on the basis of a total of 350 random points selected from the non-landslide areas (supplementary Fig. S2) resulting in a root mean square error of 0.37 m, a mean absolute error of 0.31 m in elevation, and R^2^ = 1 when considering 2012 DEM as reference data. Such accuracy is better than that were used in previous similar studies and is therefore suitable for our purpose. For instance Tang et al.^15^ by analysing landslide volumes using mult-source DEMs concluded that high resolution DEMs provides more realistic estimates than the low resolution ones, and those with larger bias.

**Figure 2:**
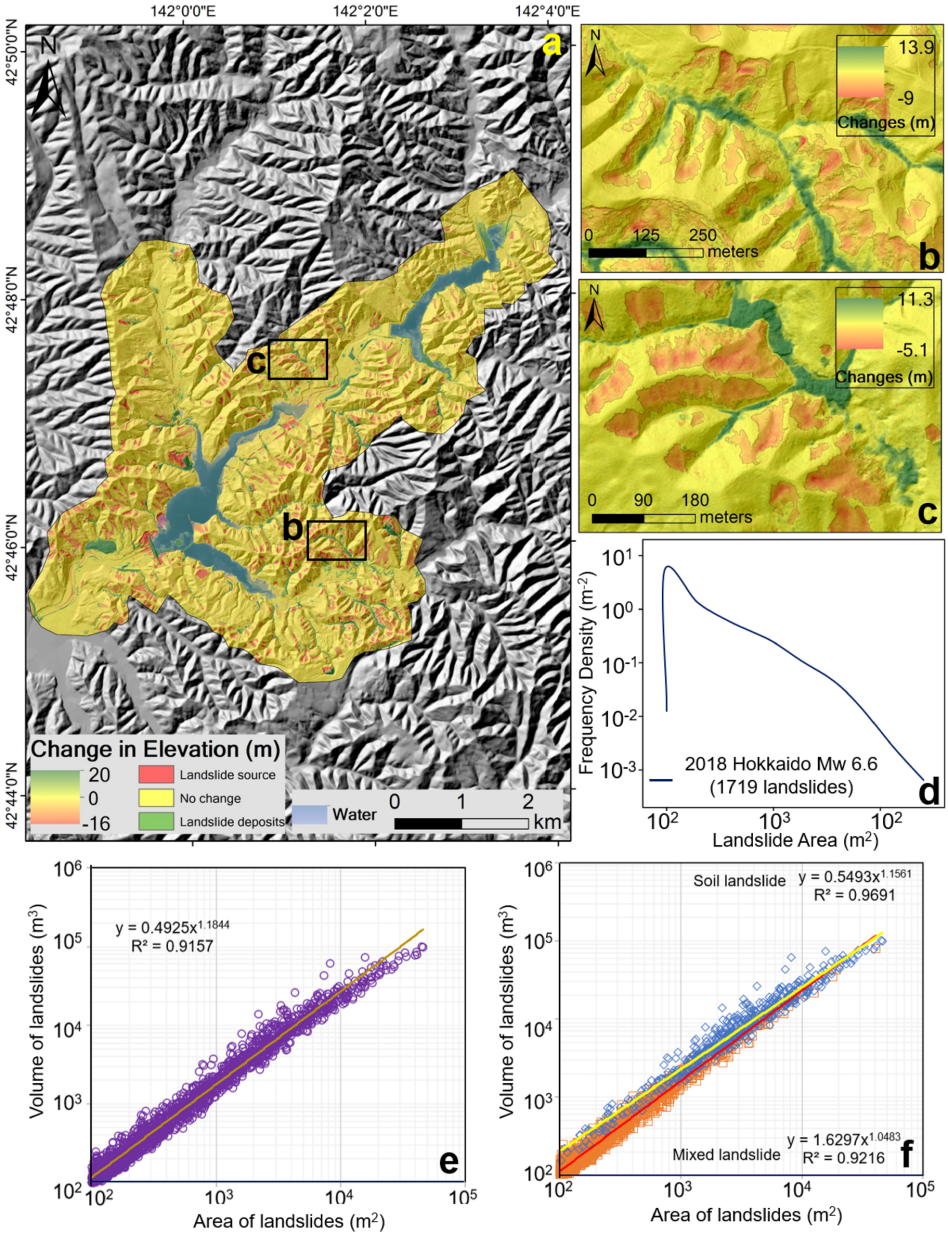
(**a**) Post and pre-event LiDAR DEM subtraction map showing changes in elevation values caused by landslides; red shows areas of landslide source and green shows deposition zones. (**b**) and (**c**) are the enlarged areas of rectangles shown in (**a**). (**d**) Shows the frequency density—landslide area plot for the 1719 landslides mapped by DEM subtraction. Correlations between landslide area and landslide volume for (**e**) all 1719 landslides and (f) shows the distinction in correlation values between soil landslides and mixed soil–bedrock landslides.

While the three available landslide inventories are used for quantifying the total landslide volume, we additionally created a new database of landslides in the calibration area (i.e., the 29.41 km^2^ coverage area of pre-earthquake LiDAR data) for developing a specific area-volume relationship. This database is created by the DEM subtraction method and identifies areas with negative changes in the elevation. By automated GIS techniques (Fig. S3, supplementary file), these negative-change areas are then mapped as landslide source zones and extracted for the area and volume analysis (Fig. 2). The automatically extracted landslides are manually cross verified with the aerial photographs (0.5 m; taken in 2018) for any inaccuracy, and if found they are manually edited in a GIS. As many as 50 landslides were visited in the field to observe the hillslope debris and to validate DEM subtraction figures.

A laser range finder (Nikon coolshot 40i), was pointed to the side walls and crown portions of landslide body to approximate the depth values. Because of the relatively small relief (average = 31 m and maximum = 201 m), almost all the landslide debris has been removed to the valley bottom. The main geometrical features (length and width) of newly mapped landslides from DEM subtraction method were extracted by specifying minimum convex hull bounding geometry enclosing the landslide polygon. Length and width are then estimated from the longest and shortest distance between any two vertices of the convex hull (Taylor et al., 2018). The area of landslides is calculated by means of the *Calculate Geometry* tool that populates the geometric values derived from landslide polygon features based on data frame's coordinate system in ArcGIS. The volumes and depths of these landslide source areas are computed by spatially aggregating sums of DEM-subtraction pixels within the landslide polygons using the following equation:

1 $$V= A{\sum }_{x=1}^{n}S$$

where V is the volume, A is the cell area in 3D, S is the subtracted value from pre-event DEM and post-event DEM for each cell, and n is the cell count.

Further, to examine the landslide area-volume relationship, a relative volume-area scaling for the landslides in the study area was assessed based on the following equation^22^:

2 $$V= \alpha {A}^{\gamma }$$

where V is the landslide volume, A is the landslide area, α is the intercept, and γ is the scaling exponent. To investigate and discuss the uncertainties in existing scaling relationships and their influence on total volume estimates, we then compare the results of our area-volume relationships obtained using the newly mapped 1719 landslides from the calibration area, with existing scaling coefficients available in the literature (Table 1).

**Table 1:** Landslide area and volume scaling relationships available in the literature.

Dataset	Log10 $\alpha$	$\gamma$	R^2^	n	References
Global	− 0.836 ± 0.015	1.332 ± 0.005	0.95	4,231	^22^
Global	− 1.131	1.45 ± 0.009	0.97	677	^36^
Global bedrock	− 0.73 ± 0.06	1.35 ± 0.01	0.96	604	^22^
Global soil	− 0.44 ± 0.02	1.145 ± 0.008	0.90	2136	^22^
Japan soil	− 0.41	1.31	0.84	51	^21^
Japan soil	− 0.72	1.19	0.86	11	^37^
Japan mixed	− 0.60 ± 0.06	1.36 ± 0.03	0.88	236	^22^
Himalayas soil	− 0.44 ± 0.07	1.25 ± 0.03	0.92	141	^22^
Himalayas mixed	− 0.59 ± 0.03	1.36 ± 0.01	0.98	428	^22^
Himalayas bedrock	− 0.49 ± 0.08	1.34 ± 0.02	0.98	123	^22^
New Zealand (all)	− 0.86 ± 0.05	1.36 ± 0.01	0.97	389	^22^
New Zealand (all)	− 0.05	1.109	0.68	8442	^12^
New Zealand soil	− 0.37 ± 0.06	1.13 ± 0.03	0.86	237	^22^
New Zealand soil	0.12	1.060	0.69	1824	^12^
New Zealand Mixed	− 0.86 ± 0.05	1.36 ± 0.01	0.97	389	^22^
New Zealand bedrock	− 0.13	1.138	0.67	6618	^12^
China, Wenchuan	− 0.975	1.388	–	41	^3^
China, Wenchuan	0.119	1.209	0.78	1415	^20^
Korea	− 0.229	1.02	0.89	930	^38^

Lastly, to compare co-seismic landslide-induced volume losses with tectonically driven volume gains in the landscape, we establish uplift rates produced by the M_w_ 6.6 Hokkaido Eastern Iburi Earthquake for the study area using the following method. GSI estimated the ground displacement caused by the earthquake and modelled the fault slip position by employing ALOS-2 SAR (DAICHI-2) data acquired before and after the earthquake together with data from the global satellite navigation system (GNSS) (GSI, 2018). We georeferenced the published vertical component map and interpolated the uplift values obtained from 11 GNSS stations (co-seismic uplift rate ranging from 0 to 3.65 cm) using the inverse distance weighted (IDW) technique (Supplementary Fig. S4), and calculated the net uplift volume caused by this M_w_ 6.6 earthquake. It is worth mentioning that our calculation of net uplift volume only covers the size of post-earthquake DEM (Fig. S3). The volume of uplift is calculated by using Parker et al. formula:

3 $${V}_{u}= A{\sum }_{x=1}^{n}{U}_{x}$$

where V_u_ is the net uplift volume, A is the cell area, U_x_, the vertical displacement for each cell, and n is the cell count.

## Results

A total of 1719 landslides are mapped in the calibration area. Area and volume of landslides vary widely in the calibration area. To minimise uncertainties in the landslide area mapping and volume estimation, we considered the smallest landslide area as 100 m^2^ in the analysis. The frequency density plot, as expected, exhibits characteristic power-law scaling (Fig. 2f). The largest mapped landslide in calibration area is 45,978 m^2^, the mean is 1995 m^2^ and the standard deviation is 4166 m^2^. The smallest landslide volume mapped in calibration area is 84 m^3^ and the largest is 10 × 10^4^ m^3^. The mean mapped volume is 4611 m^3^, with a standard deviation of 10,138 m^3^. The range of landslide depth varies from 0.45 to 24.42 m, with a mean depth of 1.72 m. The total landslide area and volume in the calibration zone are 3.43 × 10^6^ m^2^ and 7.92 × 10^6^ m^3^, respectively. It is noteworthy to mention that the areas with positive changes (3.30 km^2^, i.e., the landslide deposits) nearly equals the areas with negative changes (3.43 km^2^). We presume that a small part of debris materials might have been washed out of the catchment.

Geometrical properties such as landslide area (A), the maximum length of landslide (L), the maximum width of landslide (W), and maximum depth (H) for the 1719 landslides are correlated with the mapped volume obtained from DEM subtraction and focal statistics method. Log-transformed regression curves for the correlation plot are presented in (Fig. 2, and Supplementary S5). The R^2^, i.e., coefficients of determination for the landslide area and volume, length and volume, width and volume, and depth and volume are 0.91, 0.78, 0.78, and 0.19, respectively. As expected, the best fit correlation is obtained for landslide area and volume (Fig. 2).

### Landslide area-volume relationship

**Figure 3:**
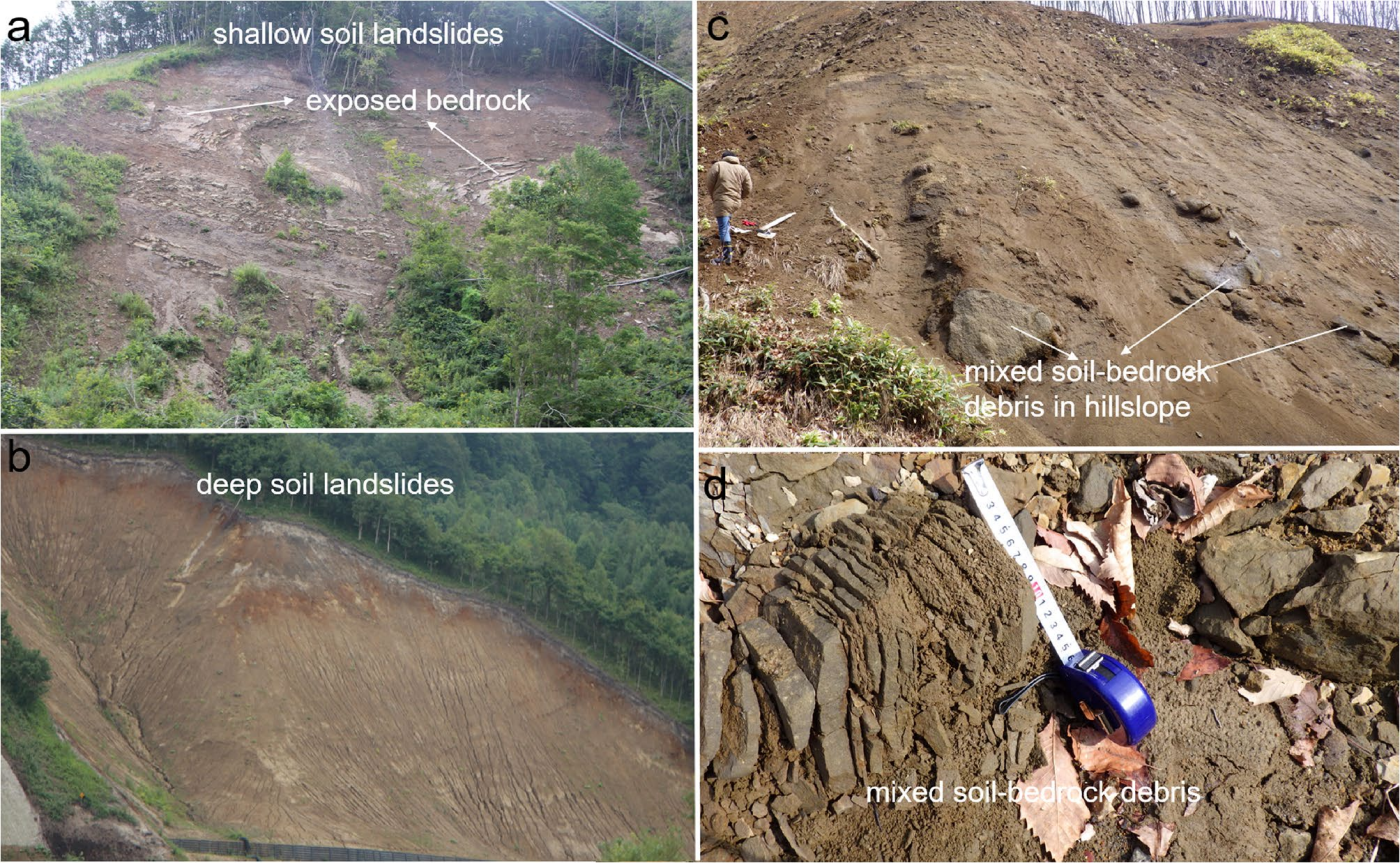
(**a**) Shallow soil landslides exposing bedrock in steep slopes, (**b**) deep soil landslides, (**c**) debris with fragments of boulders in lower hillslopes, and (**d**) mixed soil bedrock debris in the valley.

The landslide area-volume scaling relationship for the 1719 landslides in the study site is defined as V = 0.49 × A^1.18^ (R^2^ = 0.91; N = 1719) (Fig. 2). Based on our field surveys, and published literature^39,40^, most EQILs are classified as shallow soil landslides (supplementary Fig. S6). Only a few landslides have failure surfaces that have exposed bedrocks and soil layers that extend deep (Fig. 3)^32–34^. The slip surface of these shallow landslides is either the weathered tephra layer or the basement sedimentary complex. Since the failure surface in the study area is about 2.5–3.5 m in most cases, we extracted the landslides having a depth less than 3.5 m (n = 1187), and considered them as purely soil landslides. The remaining landslides (n = 532), with a depth ranging between 3.50 and 24.12 m, are classified as mixed soil–bedrock landslides. The scaling relationship for soil and mixed landslide from area-volume analysis therefore are V = 0.54 × A^1.15^ (R^2^ = 0.97) and V = 1.62 × A^1.04^ (R^2^ = 0.92), respectively (Fig. 2).

### Volume of debris generated during the Hokkaido earthquake

Based on the area-volume scaling relationship obtained from the calibration area, we estimate the total volume of earthquake-induced landslide for the Hokkaido Eastern Iburi earthquake for the three different inventories. The estimated total volume of debris for the Hokkaido Eastern Iburi earthquake, derived based on the Wang et al.^28^ inventory, is 56.17 million m^3^ for soil landslides and 64.75 million m^3^ for all landslides, whereas Dou et al.^27^ inventory give a volume of 64.72 million m^3^ and 75.61 million m^3^, respectively. The largest volume was obtained with the GSI (2018) inventory, i.e., 157.87 million m^3^ and 197.72 million m^3^.

## Discussion

Based on the landslide area-volume relatioships, we estimate that approximately 56–197 million m^3^ of debris materials were removed from the low-relief Hokkido-Iburi region after the earthquake. Estimates of the volume of landslides associated with the Wang et al.^28^ mapping are about 8.55–10.86 million m^3^ lower than Dou et al.^27^, while those mapped by GSI are about two times higher (~ 93–122 million m^3^) than Dou et al.^27^, a difference that may be attributed at least in part to mapping uncetainity. The large differences in estimated volumes demonstrate that landslide areas not divided into their source and deposition areas can strongly produce bias in volume estimates, as the relationship linking area to volume is not linear. Additionally, the degree of accuracy in the separation of multiple overlapping landslide boundaries on the same slopes, i.e., the ability to differentiate individual landslides from clustered landslides may also lead to a significant improvement in the volume estimate (Fig. S1). For example, Massey et al. (2020) reported that the amalgamation of small landslides into a single one can inflate volume by up to a factor of three.

We notice that the volume of landslides calculated from the DEM subtraction method (i.e., 7.92 × 10^6^ m^3^) in the calibration area has a close match with the Dou et al. (2020) landslide inventory (7.47 × 10^6^ m^3^) than with the other two inventories (GSI = 14.88 × 10^6^ m^3^ and Wang = 6.54 × 10^6^ m^3^). The highest accuracy found in Dou et al.^27^inventory may be attributed to their mapping pattern as their database is the only one that separates source areas from deposition areas, reducing the amalgamation bias. Therefore, this inventory has been used in the uncertainty analysis of existing scaling relationships.

### Uncertainties in the existing scaling relationships

**Figure 4:**
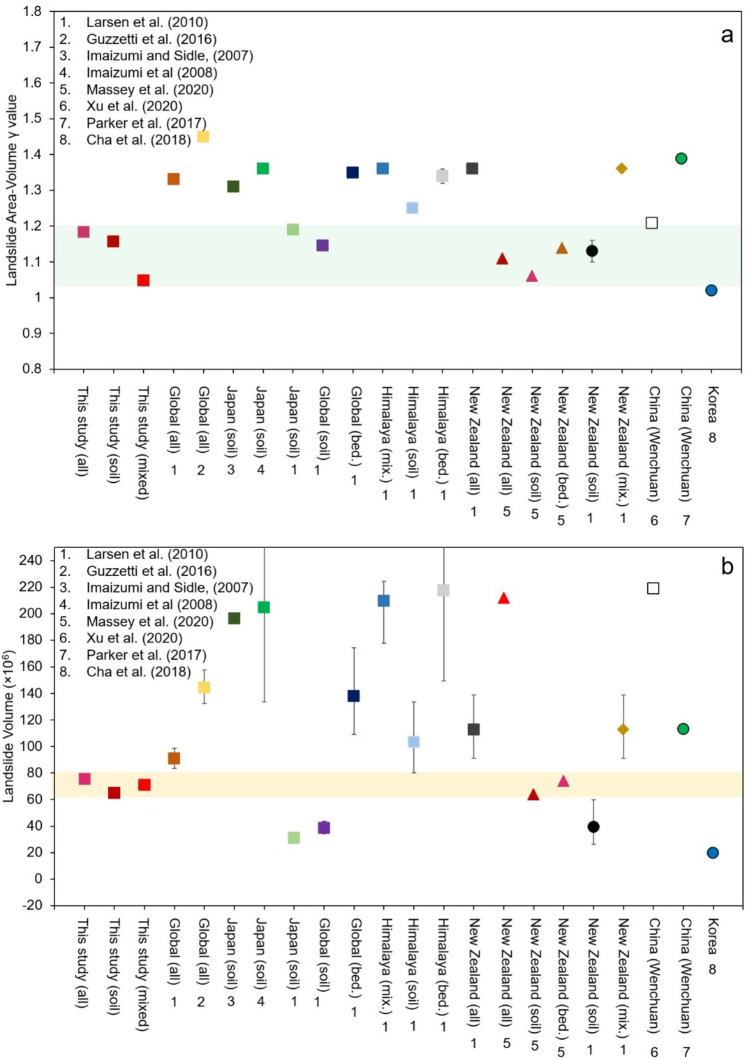
**(a)** Landslide area—volume scaling exponents (γ) obtained from this study and those studies listed in Table 1. **(b)** shows the total volume of debris produced by the Hokkaido Eastern Iburi Earthquake estimated from the new area-volume relationship obtained in this study and those listed in Table.

The preliminary estimate of the Ministry of Land, Infrastructure, Transport, and Tourism of Japan indicates that the total volume of debris generated by the 2018 Hokkaido Eastern Iburi Earthquake is about 30 million m^3^^41^. Our results suggest that the total debris volume triggered by the M_w_ 6.6 quake is anywhere between 64 and 75 million m^3^ (Fig. 4b); suggesting that the initial estimates underrated the total sediment volume from the affected catchments^41^ However, such large uncertainties may have consequences in a post-hazard scenario setting. For instance, we noticed that a few check dams constructed in the region to arrest the sediment flow were full within 3 years after the event (see supplementary Fig. S7). As an example, during the Wenchuan earthquake, the landslides produced approximately 2.6 × 10^9^ m^3^ of debris deposited on the hillslopes and valleys in the entire affected area (~ 13,800 km^2^) ^3,42^, providing abundant sources for debris flows.

A decade after the Wenchuan earthquake, catastrophic debris flows remain a looming threat in the Wenchuan catchments^43^. Therefore, reliable estimates of sediment volume still available on slopes are a necessary first step required for the development of post-hazard mitigation strategies based on post-seismic residual risk, as well as for calibrating sediment budget models. Since area mapping of landslides can be easily and quickly done with the help of optical remote sensing techniques^43,44^, a quick workaround for volume estimation based on an accurate scaling coefficient is the optimal approach for most uses. To better ascertain the degree of accuracy of our empirical relation, we compared the existing models on volume determination with our results based on Dou et al. (2020) inventory (Fig. 4).

The coefficient α obtained for soil landslides in this study is 0.49–0.55, whereas the exponent γ is 1.15–1.18. The γ values of 1.15–1.18 indicate that the depth of the landslides in the region is almost uniform, which is confirmed by our field observations. In other soil landslide area-volume relationships^21,22,37^, a smaller α coefficient is noticed (0.19– 0.48), whereas γ values widely range from 1.11 to 1.31, with an average of 1.18, indicating that γ values obtained in this study for landslides initiated by the Hokkaido Eastern Iburi earthquake are well within the range reported in the literature (Fig. 4a). On the other hand, the α coefficient obtained for soil landslides mapped for New Zealand that used a high-resolution dataset for volume mapping was found to be on the higher end (α = 1.31) with a lower γ value (γ = 1.06)^12^. However, despite the large difference in α and γ, the total volume of landslides mapped for Hokkaido earthquake estimated from this study and the area-volume relationship of Massey et al.^12^are found similar to each other (Fig. 4b).

The significant difference in a large number of small landslides mapped in the Kaikoura inventory perhaps caused the differences in the coefficients (see Supplementary Fig. S8). All other soil landslide scaling relationships except two underestimated the volume values. The scaling relationships derived from Imaizumi and Sidle (2007) and Imaizumi et al. (2008) overestimated the volume, possibly because of the limited number of samples (n = 51 and n = 11) used in the analysis. Additionally, the landslides reported in these two studies are precipitation induced. The good match in volume values obtained for Hokkaido earthquake induced landslides from our relationship and that from Massey et al.^12^relationship could be related to the high accuracies of the mapping measurement used. Moreover, like the Hokkaido Eastern Iburi co-seismic landslides, the Kaikoura inventory also contains a high proportion of shallow soil failures^1^.

### Co-seismic net uplift and erosion volume

Apart from the mountain-building process in the form of continuous uplift, another fundamental concept of orogenic evolution is that mountains are built through recurring cycles of vertical uplifts by earthquakes^45^ This process was questioned in Parker et al.^3^, that discuss whether large earthquakes create or destroy mountainous topography. By analysing the landslides triggered by the 2008 M_w_ 7.9 Wenchuan earthquake, their study shows that co-seismic landslide volume (~ 5–15 km^3^) is about 2 to 6 times greater than co-seismic uplift volume (~ 2.6 km^3^), thus suggesting the need for careful consideration of the role of co-seismic uplift-erosion balance in topographic development. Nevertheless, the Wenchuan earthquake was one of a kind in which the seismic pulse produced the largest number and volume of co-seismic landslides in mapping history. With this notion in mind, and to validate the Parker et al.^3^findings, we compare the net uplift volume of the 2018 Hokkaido Eastern Iburi Earthquake event, and the net erosion volume caused by landslides in our testing area. The calculated net uplift volume in the study area (~ 512 km^2^: testing site) was found to be 5.55 million m^3^. One the otherhand, widespread landsliding produced 64–72 million m^3^ of debris from the affected catchments, which is found to be much greater than the estimated net uplift volume of 5.55 million m^3^. Our results seem to agree with the Parker et al.^3^results and suggest that frequent large earthquakes may counterbalance the topographic uplift through erosion by landslides. This simple straightforward analysis may have overlooked isostatic compensation, inter-seismic vertical movement, and flexural rigidity of the lithosphere^46^; hence should be interpreted with caution. Nonetheless, Hovius et al.^47^ reported that ~ 840 Mt of sediment had been moved out of the Choshui catchment in Taiwan following the Chi-Chi earthquake of 1999 until up to 2007, equaling to 4/5th of the rock mass added by the earthquake. Adding the background erosion rate in the region by rainfall and typhoons, their work also concluded that the tectonic mass flux added into the orogenic systems can be countered by landslides in a few years.

## Conclusions

The 2018 Hokkaido Eastern Iburi earthquake caused a large number of landslides in more than 500 km^2^ area. Using 1 m LiDAR elevation models, we calibrated an area-volume power-law for landslides and found that the values of γ exponent (1.15–1.18) for the 1719 predominantly soil landslides initiated by this earthquake lies within the centre of the range of γ values reported for soil landslides in the literature. However, we noticed large deviations in the total estimates of debris volume from existing area-volume relationships. We attribute this discrepancy to contrasting methods and mapping inaccuracies due to low-resolution DEMs used in previous studies. Failure to account for source and deposit areas, and the amalgamation of small landslide clusters can also significantly contribute to overestimating the total volume, leading to incorrect estimates. Results of the present study show that the net uplift produced by this earthquake deformation is smaller than the total eroded volume produced by co-seismic landslides.

## Data Availability

The airborne LiDAR-derived digital elevation models presented in the paper were originally provided by geospatial information of Hokkaido and the processed DEM files and mapped landslides are available from https://doi.org/10.6084/m9.figshare.21858687 and https://doi.org/10.6084/m9.figshare.21858726, respectively.
